# Are Cranial Biomechanical Simulation Data Linked to Known Diets in Extant Taxa? A Method for Applying Diet-Biomechanics Linkage Models to Infer Feeding Capability of Extinct Species

**DOI:** 10.1371/journal.pone.0124020

**Published:** 2015-04-29

**Authors:** Zhijie Jack Tseng, John J. Flynn

**Affiliations:** 1 Division of Paleontology, American Museum of Natural History, Central Park West at 79th Street, New York, New York, 10024, United States of America; 2 Richard Gilder Graduate School, American Museum of Natural History, Central Park West at 79th Street, New York, New York, 10024, United States of America; Team 'Evo-Devo of Vertebrate Dentition', FRANCE

## Abstract

Performance of the masticatory system directly influences feeding and survival, so adaptive hypotheses often are proposed to explain craniodental evolution via functional morphology changes. However, the prevalence of “many-to-one” association of cranial forms and functions in vertebrates suggests a complex interplay of ecological and evolutionary histories, resulting in redundant morphology-diet linkages. Here we examine the link between cranial biomechanical properties for taxa with different dietary preferences in crown clade Carnivora, the most diverse clade of carnivorous mammals. We test whether hypercarnivores and generalists can be distinguished based on cranial mechanical simulation models, and how such diet-biomechanics linkages relate to morphology. Comparative finite element and geometric morphometrics analyses document that predicted bite force is positively allometric relative to skull strain energy; this is achieved in part by increased stiffness in larger skull models and shape changes that resist deformation and displacement. Size-standardized strain energy levels do not reflect feeding preferences; instead, caniform models have higher strain energy than feliform models. This caniform-feliform split is reinforced by a sensitivity analysis using published models for six additional taxa. Nevertheless, combined bite force-strain energy curves distinguish hypercarnivorous versus generalist feeders. These findings indicate that the link between cranial biomechanical properties and carnivoran feeding preference can be clearly defined and characterized, despite phylogenetic and allometric effects. Application of this diet-biomechanics linkage model to an analysis of an extinct stem carnivoramorphan and an outgroup creodont species provides biomechanical evidence for the evolution of taxa into distinct hypercarnivorous and generalist feeding styles prior to the appearance of crown carnivoran clades with similar feeding preferences.

## Introduction

Measures of biological function and performance link morphological variation with survival and reproductive fitness, and are a means of identifying functional morphological adaptations [1]. Studying the linkages between morphological disparity and taxonomic and functional diversity is a long tradition in paleontology, as the fossil record provides crucial data on macroevolutionary patterns and processes. The nearly complete reliance on analysis of morphological characteristics that are amenable to fossilization introduces difficulties in establishing a morphology-diet linkage model between predicted functional morphology and known feeding preferences of extant species. Namely, interpretations of the paleobiology of extinct species and their evolutionary trends may be confounded by the ubiquitous phenomenon of many-to-one form-function mapping in vertebrate structures [2]. Complex biological and physical systems are exposed to multiple, simultaneous functional demands at different hierarchical levels of organization; as such, optimization of a particular trait or attribute may be compromised by trade-offs required in other traits or attributes in the same system. As a result, one biological function may be performed by morphologies taking many forms, and vice versa. Developing methods for successfully deciphering the relationships between form and function in spite of this complexity, is critical for enhancing our understanding of morphological adaptations, both in living ecological webs and through deep time, as well as how the patterns of functional innovations co-varied with diversification and extinction patterns.

Functional or adaptive significance is often assigned to similar morphological characteristics observed across unrelated lineages, in the form of potentially convergently evolved features [3]. Major examples, such as the independent evolution of wings for powered flight in birds, insects, mammals, and pterosaur reptiles, or the ecologically similar habits and morphological characteristics of arboreal, fossorial, or cursorial species within both eutherian and metatherian mammals, point to the functionality and adaptive significance of such morphological innovations in both extant and extinct species. Studies that search for consistent links between organismal form and adaptive function thus are important for interpreting past ecological diversity based on observed morphological disparity, and for better characterizing the pathway of convergent morphologies among extant species. A prominent example of convergence in eutherian mammals is the iterative evolution of ecologically important predators and of generalist consumers in the order Carnivora [4]. The more than 280 extant carnivoran species can be placed into a few broadly defined feeding preferences that are associated with unique dental and musculoskeletal characteristics. These major dietary preferences include hypercarnivores (specialists on vertebrate soft tissues; e.g., felids and large canids), durophagous hypercarnivores (specialists on vertebrate hard and soft tissues; e.g., hyaenids), herbivores (e.g., red panda and giant panda), invertebrate/vertebrate generalist feeders (e.g., some herpestids and mustelids), and omnivores (e.g., ursids, procyonids, and some viverrids) [5,6]. Evolution of this wide range of dietary preferences has resulted in carnivorans playing key roles within many modern and fossil ecological communities.

The evolutionary patterns of taxonomic diversification in Carnivora can be traced back to the early Cenozoic, when their predecessors in the stem lineages of Carnivoramorpha (of which Carnivora is the crown clade) first evolved key morphological features of extant predators, such as a single pair of shearing carnassials and crushing talonid basins in the dentition [7,8,9,10]. Therefore, a key to understanding the patterns and processes of taxonomic and ecological diversification in crown Carnivora relies on a better understanding of the functional diversity of stem carnivoramorphan morphologies. In this paper, we test how well mechanical properties of the cranium reflect correlations between craniodental morphology and dietary preferences in extant carnivorans, using species representing hypercarnivore, invertebrate/vertebrate generalist, and omnivorous feeding styles. We then test what such a diet-biomechanics relationship can reveal about the diet of extinct species when applied to fossils, such as a stem carnivoramorphan and a closely related outgroup species (the creodont *Thinocyon*).

## Materials and Methods

No permits were required for the described study. The specimens used in the study are all part of existing museum collections in the United States. Complete list of specimens used: *Canis lupus*, LACM(M)23010 (Department of Mammalogy, Natural History Museum of Los Angeles County, California, USA). *Herpestes javanicus*, AMNH(M)10165; *Mephitis mephitis*, AMNH(M)17213; *Panthera pardus*, AMNH(M)11374; *Procyon lotor*, AMNH(M)24815 (Department of Mammalogy, American Museum of Natural History, New York, USA). *Oodectes herpestoides*, AMNH140008; *Vulpavus palustris*, AMNH11497; *Vulpavus ovatus*, AMNH11498 (Division of Paleontology, American Museum of Natural History, New York, USA), *Thinocyon velox*, FMNH PM60215 (Field Museum of Natural History, Chicago, USA).

Because comparative empirical biomechanical data are not currently available for all carnivoran species, part of our goal is to generate a diet-biomechanics linkage model that can be applied to both extant and extinct species of unknown or uncertain feeding preference. We investigate form-function mapping through a combination of shape analysis using geometric morphometrics (GMM) and biomechanical simulations, with a modeling approach based on Finite Element Analysis (FEA). GMM methods permit quantitative description of shape variation based on homologous landmark coordinates or regions, in two or three dimensions (2-D or 3-D)[11,12]. FEA arose as an engineering simulation method for examining and predicting mechanical behavior in machined parts or complex structures, but more recently has been applied to study biomechanical questions across the biological sciences [13]. We used high-resolution x-ray micro-computed tomography (HRXμCT) to capture 3-D morphological data for the carnivoramorphan species on which GMM and FEA methods were applied.

**Fig 1:**
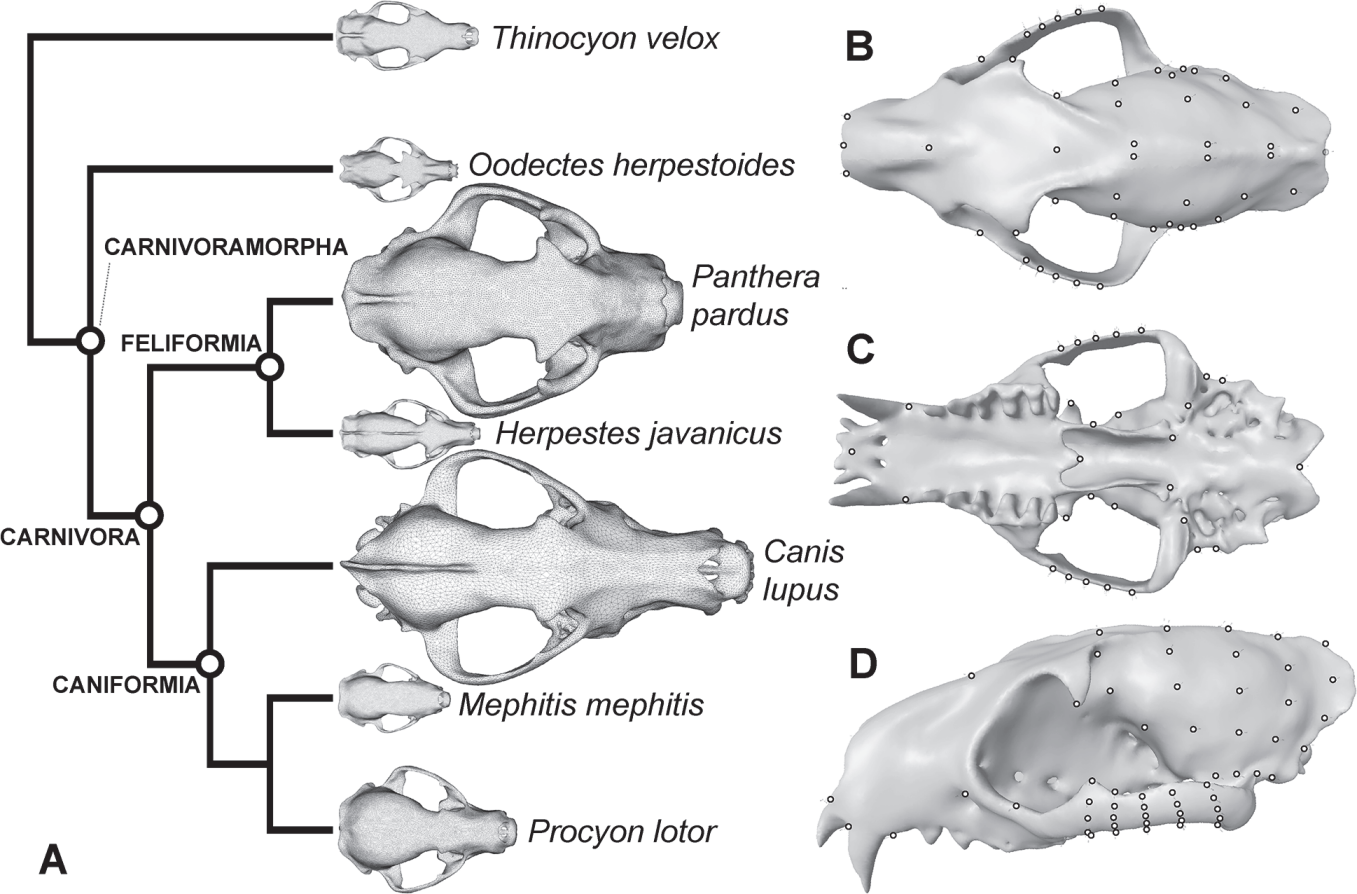
Species, relationships, and anatomical landmarks used for FE and GMM analyses. **A**. Phylogenetic relationships for the species sample [56], with names of more inclusive clades indicated at internal nodes. The outgroup species is the creodont *Thinocyon velox*; creodonts are close relatives of Carnivoramorpha, together forming the Ferae [50]. **B**. Dorsal view of anatomical fixed and semi-landmarks. **C**. Ventral view of landmarks. **D**. Dorso-lateral view of landmarks on a generic skull representing the average shape of species examined.

Five extant carnivorans, 1 extinct carnivoramorphan, and an out-group creodont species were sampled for these analyses. Extant species included two hypercarnivores (species that consume vertebrate soft and hard tissues as their main food source, usually accompanied by specializations of the teeth and skull): *Canis lupus* (gray wolf) and *Panthera pardus* (leopard), two invertebrate/vertebrate generalist feeders (species that consume a mix of invertebrate/vertebrate animals as their main food source): *Mephitis mephitis* (striped skunk) and *Herpestes javanicus* (small Asian mongoose), and an omnivore (species without a clear dietary preference for animals versus plants): *Procyon lotor* (raccoon). The exemplar species across these three major feeding groups were selected to span the broad size range of terrestrial carnivorans, and to include representatives from both caniform and feliform clades (the two major branches of Carnivora), as well as from different families within those two clades (Fig 1). Furthermore, we chose two extreme examples of hypercarnivory (pursuit hunting *Canis* and ambush hunting *Panthera*) to partially account for known behavioral and morphological diversity of extant taxa categorized as hypercarnivores. The stem carnivoramorphan *Oodectes herpestoides*, represented by a nearly complete skull [9] and the best preserved among all known extinct carnivoramorphans in overall completeness and lack of deformation, was included to test dietary preference assignment based on extant form-function relationships. Lastly, an extremely well-preserved specimen of the small-bodied creodont *Thinocyon velox*, represented by a nearly complete and undeformed skull, was included as the outgroup comparator for Carnivoramorpha.

### Finite Element (FE) Analysis

All seven specimens, except for *Canis lupus*, were scanned using a GE v|tome|x HRXμCT at the Microscopy and Imaging Facility (MIF) of the American Museum of Natural History (AMNH), at a voltage of 150–170 kV, tube current of 55–180 mA, and a voxel size of 37.69–136.00 μm. Digital image data for *C*. *lupus* were from the prior study of Tseng [14]. The raw X-ray data were reconstructed in Phoenix Datos (GE Measurement and Control, USA) and VG Studio Max (Volume Graphics GmbH, Germany) into stacks of coronal section images, and then imported into Mimics (Materalise, Belgium), in which a 3-D surface mesh was generated. Incomplete zygomatic regions in *O*. *herpestoides* were reconstructed using a digital section of the zygomatic of *H*. *javanicus* as a starting point because the latter most closely resembled *O*. *herpestoides* in size and superficial morphology among the taxa studied. Then the reconstructed model was morphed in Geomagic Studio (Geomagic Inc., USA) based on the zygomatic fragments belonging to the same fossil specimen of *Oodectes*, and incorporating additional comparisons to the zygomatic morphology of stem carnivoramorphans *Vulpavus palustris* (AMNH11497) and *V*. *ovatus* (AMNH11498). Assuming bilateral symmetry, the skull of *T*. *velox* was reconstructed digitally, and the more complete right side was mirrored to create a symmetrical specimen. The right zygomatic arch of the skull was diagenetically deformed medial-dorsally, with breaks at both anterior and posterior connections to the cranium, and therefore was digitally rotated ~15° back into the estimated original position.

The skull surface meshes then were re-meshed in 3-matic (Materialise, Belgium) to control for geometric error and improve triangle element aspect ratios (set to 1:6 min:max aspect). The triangle meshes then were imported into Strand7 FEA software version 2.4.6 (G+D Computing Pty Ltd, Australia) and converted into finite element mesh models using 4-noded tetrahedral elements. Because finite element densities have varying effects on solution results in the modeling protocol [15], we used a previously tested resampling protocol of building 3 finite element models ranging from ~500k to 1,500k tetrahedral elements, and then using model means and standard error ranges for comparison of results among species.

We simulated unilateral bites across the upper dentition (from canines to the most posterior tooth, which varies across the clade in specific locus of the last tooth) in each species model set, taking average values from analyses of the left and right dentitions. Symmetry of the skull is assumed, but not necessarily of the finite elements making up the models, so left-right averages are used to account for asymmetry in results caused by asymmetric placement of finite elements. Input forces for the working (biting) side temporalis and masseter muscles were calculated by multiplying estimated physiological cross-section area (ePCSA) obtained using the dry skull method [16,17] and the maximum tension produced by mammalian muscle fibers (0.3 N/mm^2^; [18]). Because species-specific data on pterygoid ePCSA are not available, and a previous survey of pterygoid mass in carnivorans provided a range of 8–13% of total jaw-closing muscle mass [19,20], we included an estimated 10% contribution (input force sum of temporalis+masseter being 90% of total) of the pterygoid muscles in all species models, to incorporate this relatively small but nonetheless biomechanically contributing jaw-closing muscle group. The balancing-side muscle forces were adjusted to 60% of the estimated input force on the working side, to account for observed differences in EMG activation patterns during biting in extant carnivoran species [21,22]. Calculated input muscle forces were distributed over the attachment regions of the respective muscles using the “tangential forces” option in the program BoneLoad [23], based on anatomical study of muscle attachment sites and analogous areas in extinct species, using an average gape of 30° for comparison across the sample. Mandibles of each species were used to orient the directions of the muscle force vectors from the cranial attachment sites.

Nodal constraints were placed at each tooth position to fix the cusp tip from movement, simulating contact with a food item. Nodal constraints also were placed at each of the temporomandibular joints (TMJ), allowing rotation of the cranium only around the axis of the joint, and no rotation or displacement in any other direction. Homogeneous material properties, with Young's Modulus of 20 GPa and Poisson's ratio 0.3, were used in all models [24]. Because fossil specimens can have diagenetically modified material densities and distributions, we used homogeneous models to compare extant and fossil species. Heterogeneous models with multiple material properties assigned using density values (Hounsfield units) from the CT data have been shown to provide broadly similar patterns of stress distribution as homogeneous models; validated strain data also confirm the validity of using homogeneous models for comparative inferences of stress distribution and biomechanical capability [25,26,27].

All low- and medium- resolution models were solved in linear static analyses, in Strand7 using the direct sparse solution scheme. As the highest resolution models exceeded the maximum matrix storage size allowed by the program (42 GB), the conjugated gradient solver was implemented with 10,000 iterations. The parameters used for comparison among models included the output bite force measured from the nodal constraint at the respective tooth positions (in Newtons, N), the mechanical efficiency (ME) or the ratio of input load to output bite force, total strain energy (SE) in the skull (a measure of the work done by a structure under load, with higher magnitudes indicating lower stiffness in the structure for a given load), and von Mises stress distributions on the surface of the skull models [28]. We use these measures as parameters to correlate diet and biomechanics because 1) the magnitude of bite force and the efficiency with which it is produced directly determine the range of food material properties that can be masticated (harder food requires higher bite forces), and 2) skull strain energy is a measure of skull stiffness, and stiffer skulls that transmit more input muscle force into output bite force as work done on food during mastication should be favorably selected. Stiffer skulls with higher efficiency of mastication also should be associated with lowered risk of damaging cranial bones that protect sensory organs from stresses and strains that pass through the cranial region during mastication. 3) von Mises stresses have been used as failure criteria for materials such as bone that undergo a ductile mode of deformation, and applied to bone simulations to identify regions most likely to fail under biological loading conditions [24, 28]. It is important to note that bite forces predicted from the FE models are estimated values; the forces should be used for relative comparison only as they may represent underestimates of *in vivo* bite forces [17]. All biomechanical models analyzed in this study are available at the Dryad data repository (doi:10.5061/dryad.1b52s).

### Geometric morphometric analyses

The FE tetrahedral mesh models were exported as files in stereolithography format, and 3-D landmarks were digitized in Landmark editor [29]. Thirty-eight fixed, homologous anatomical landmarks and four semi-landmark surface-patches (total of 88 semi-landmarks, in addition to fixed landmarks, for a sum of 136 landmarks) were placed on each of the species models (Fig 1 and Table 1). Three alignment methods were used to account for different treatments of semi-landmarks in creation of three distinct datasets:

1. A Procrustes superimposition analysis, treating all landmarks as fixed, was used to align the landmarks in MorphoJ [30], and a principal components analysis (PCA) was conducted on the covariance matrix of the landmarks, using the residuals of a linear regression between landmark variables and log centroid size.
2. Surface-patch semi-landmarks were allowed to slide, and were first aligned using minimized bending energy in the R package Geomorph [31], and the dataset then was aligned using the Procrustes method in MorphoJ.
3. Semi-landmarks were aligned using Procrustes superimposition first, in Geomorph, then the entire dataset was analyzed in MorphoJ.

**Table 1:** Anatomical landmarks and surface semi-landmarks used in GMM analyses.

No.	Description
1	Mid-sagittal premaxillary suture at dorsal base of I1 crown
2	Mid-sagittal nasal suture, anterior-most edge
3	Mid-sagittal position of anterior rim of orbit from dorsal view
4	Mid-sagittal position of postorbital constriction
5	Posterior point of sagittal crest
6	Midsagittal ventral border of foramen magnum
7	Posterior mid-sagittal border of palate
8	Mid-sagittal premaxillary suture at ventral base of I1 crown
9	Inflection point of the narial ridge of the premaxilla from lateral view (right side)
10	Anterior border between the maxilla and canine (right side)
11	Rostral border of the first tooth in the cheek dentition at the ventral surface of the maxilla (right)
12	Rostro-dorsal border of the infraorbital foramen (right side)
13	Ventral-most point of the orbital rim edge (right side)
14	Tip of the postorbital process of the frontal (right side)
15	Tip of the postorbital process of the jugal (right side)
16	Base of the zygomatic arch at the caudal-facing face of the maxilla (right side)
17	Inflection point of the palate and the pterygoid process (right side)
18	Inflection point of the pterygoid process and the ventral-facing basicranial plane (right side)
19	Rostral border of the medial origin of the zygomatic arch on the squamosal (right side)
20	Lateral-most point of the zygomatic arch, at its dorsoventrally centered point (right side)
21	Medial base of the glenoid ridge/process (right side)
22	Caudal border of the medial origin of the zygomatic arch on the squamosal (right side)
23	Rostral base of the occipital condyle (right side)
24	Inflection point of the narial ridge of the premaxilla from lateral view (left side)
25	Anterior border between the maxilla and canine (left side)
26	Rostral border of the first tooth in the cheek dentition at the ventral surface of the maxilla (left)
27	Rostro-dorsal border of the infraorbital foramen (left side)
28	Ventral-most point of the orbital rim edge (left side)
29	Tip of the postorbital process of the frontal (left side)
30	Tip of the postorbital process of the jugal (left side)
31	Base of the zygomatic arch at the caudal-facing face of the maxilla (left side)
32	Inflection point of the palate and the pterygoid process (left side)
33	Inflection point of the pterygoid process and the ventral-facing basicranial plane (left side)
34	Rostral border of the medial origin of the zygomatic arch on the squamosal (left side)
35	Lateral-most point of the zygomatic arch, at its dorsoventrally centered point (left side)
36	Medial base of the glenoid ridge/process (left side)
37	Caudal border of the medial origin of the zygomatic arch on the squamosal (left side)
38	Rostral base of the occipital condyle (left side)
39–59	Left temporalis muscle attachment region, anchored by landmarks 4, 5, 34, 36, 37
60–80	Right temporalis muscle attachment region, anchored by landmarks 4, 5, 19, 21, 22
81–103	Left masseter muscle attachment region, anchored by landmarks 30, 35
104–126	Right masseter muscle attachment region, anchored by landmarks 15, 20

Landmarks 1–38 are fixed landmarks, 39–126 are surfaces with semi-landmarks.

A phylogeny, with topology based on Wesley-Hunt and Flynn [9] and Spaulding and Flynn [10], was imported into MorphoJ, and mapped onto the morphospace generated by PCA of each of three aligned datasets. Regression analyses between shape and biomechanical variables were conducted both on the original variables and using phylogenetic independent contrasts (PIC) calculated in MorphoJ and the PDAP module of Mesquite [32].We set all branch lengths equal to 1, and used the PDAP diagnostic tool to test for significant correlation between standardized PICs and their standard deviations [33].The lack of a significant correlation indicates that the tree topology and equal branch length used in our analyses are adequately fitted to the continuous terminal node data examined. Furthermore, uncertainties in branch lengths have only a limited effect on the analyses done using PICs [34]. The use of uniform branch lengths in phylogenetic comparative methods is commonly referred to as a speciational Brownian motion model [35].

## Results

### Finite Element (FE) Analysis

**Fig 2:**
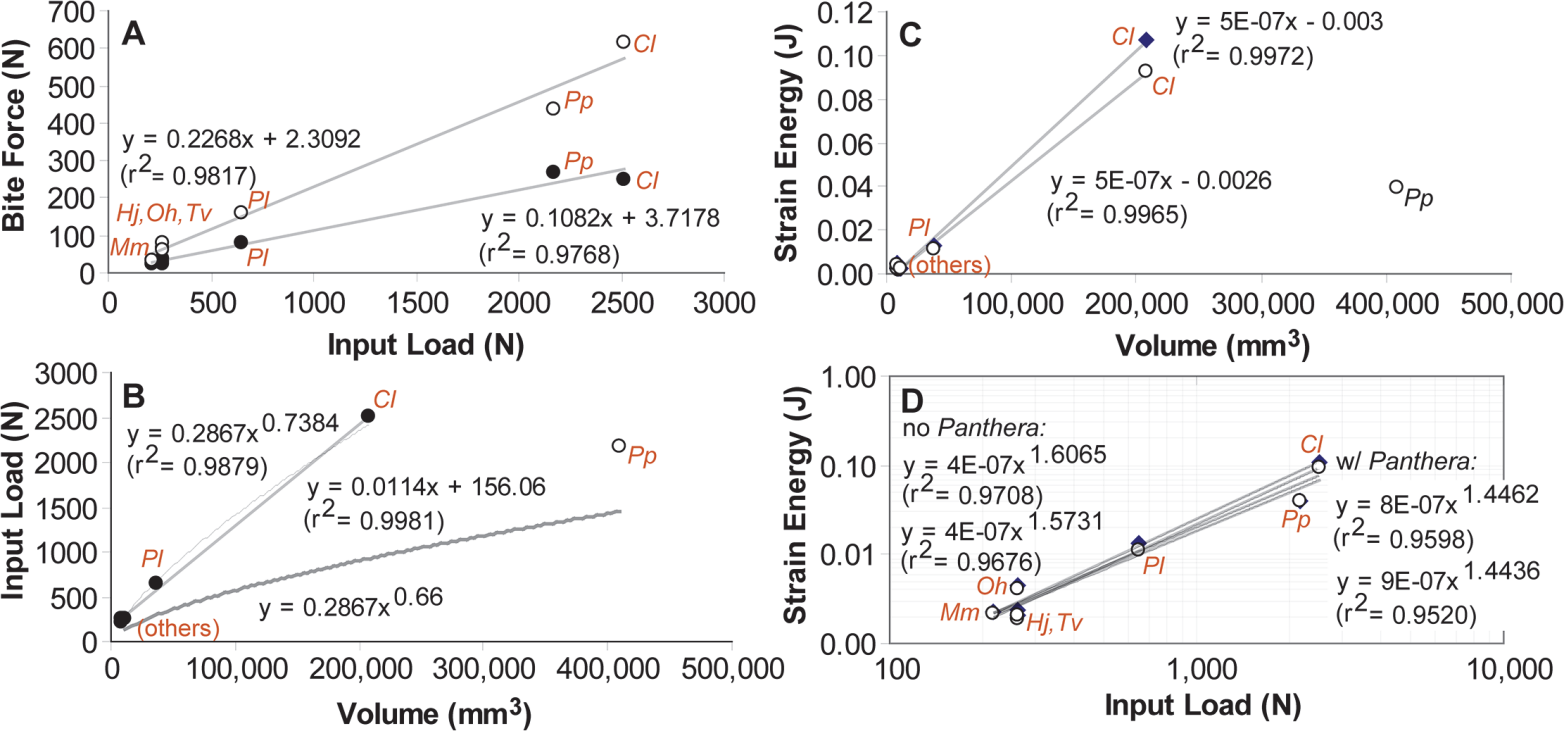
Regression analyses of FE analyses on upper dentitions. Results bracketing the entire dentition are shown by species values at the first tooth position (dark circles) and the last tooth position (hollow circle) for parts A, C, and D.**A**. Output bite force versus input load, in Newtons. **B**. Input load versus total model volume, circles represent species values, with the outlier *Panthera pardus* indicated by a hollow circle. The two upper fitted curves represent exponential and linear regressions without the outlier. The bottom curve shows regression incorporating the outlier. **C**. Total strain energy versus total model volume. Both regressions did not include the outlier. **D**. Strain energy versus input load. Regression analyses were conducted with and without *Panthera pardus* because that species has outlying values for both input load and strain energy estimates. The last tooth position (hollow circles) may be a different locus in taxa sampled, ranging from M1 to M3, because of evolutionary loss of teeth at the posterior end of the tooth row. Abbreviations: *Cl*, *Canis lupus; Hj*, *Herpestes javanicus; Mm*, *Mephitis mephitis; Oh*, *Oodectes herpestoides; Pl*, *Procyon lotor; Pp*, *Panthera pardus; Tv*, *Thinocyon velox*.

Results of FE analyses show an isometric relationship between input load and output bite force (Fig 2A and Table 2); this trend is significant in both regression analyses of the raw values and phylogenetic independent contrasts (PICs). Regression analyses also show a positively allometric relationship between input load and volume, and isometry between strain energy and volume (Fig 2B and 2C). However, neither trend was statistically significant in regressions using PICs (Table 3).

**Table 2:** Summary of FEA (n = 264 analyses).

MODEL		Lores				Medres				Hires			
SIDE		Left		Right		Left		Right		Left		Right	
ATTRIBUTE		BF	SE	BF	SE	BF	SE	BF	SE	BF	SE	BF	SE
*Canis lupus*	C	242	0.092	267	0.096	251.5	0.115	248.2	0.109	241.1	0.115	248.4	0.115
	P1	287	0.094	288	0.089	274.2	0.109	268.3	0.104	268.9	0.109	263.5	0.107
	P2	334	0.099	331	0.089	316.9	0.109	309.1	0.1	308.5	0.11	301.1	0.103
	P3	369	0.089	384	0.086	362.1	0.1	373	0.098	349.6	0.102	349.4	0.1
	P4	461	0.092	454	0.083	436.1	0.1	423.9	0.093	423.3	0.1	415	0.096
	M1	556	0.094	562	0.076	530.9	0.094	522.9	0.086	512	0.089	511.7	0.087
* *	M2	463	0.087	685	0.099	650.3	0.084	645.5	0.121	625.4	0.081	628.7	0.084
*Herpestes javanicus*	C	30.4	0.002	30.9	0.002	28.22	0.003	28.23	0.003	29.65	0.002	29.67	0.003
	P1	34	0.002	34.7	0.002	31.54	0.002	31.79	0.003	33.19	0.002	33.39	0.002
	P2	37.5	0.002	38.2	0.002	34.65	0.003	34.59	0.003	36.6	0.003	36.44	0.002
	P3	43.2	0.002	43.2	0.002	39.38	0.003	39.49	0.003	41.3	0.002	41.41	0.002
	P4	48.7	0.002	49.6	0.002	45.36	0.002	45.34	0.002	46.92	0.002	47.11	0.002
	M1	18.5	0.002	58	0.002	52.94	0.002	53.03	0.002	55.44	0.002	55.45	0.002
* *	M2	66.5	0.002	67.4	0.002	60.22	0.002	60.6	0.002	63.36	0.002	63.36	0.002
*Mephitis mephitis*	C	22.7	0.002	21.4	0.002	23.45	0.002	22.87	0.002	20.48	0.003	20.48	0.003
	P2	27.8	0.002	24	0.002	26.37	0.002	25.42	0.002	22.8	0.003	22.8	0.003
	P3	28.8	0.002	25.1	0.002	28.31	0.002	26.85	0.002	24.25	0.003	23.91	0.003
	P4	30.7	0.002	29.4	0.002	32.08	0.002	30.94	0.002	27.15	0.003	28.18	0.003
* *	M1	36.1	0.002	33.9	0.002	37.21	0.002	36.53	0.002	31.76	0.003	32.2	0.003
*Oodectes herpestoides*	C	35.5	0.004	35.5	0.004	36.73	0.005	35.97	0.005	39.72	0.005	39.47	0.004
	P2	42.9	0.004	43.1	0.004	44.56	0.004	43.71	0.005	48.05	0.004	47.8	0.004
	P3	48.3	0.004	48.3	0.004	49.64	0.004	48.75	0.005	54.31	0.004	53.88	0.004
	P4	53.8	0.004	53.9	0.004	56.23	0.004	55.21	0.005	60.02	0.004	60.54	0.004
	M1	60.3	0.004	60.8	0.004	63.19	0.004	62.31	0.005	68.14	0.004	68.5	0.004
	M2	67.4	0.003	68.6	0.004	71.49	0.004	70.75	0.005	76.29	0.004	76.62	0.004
* *	M3	74.1	0.003	75.1	0.004	77.99	0.004	77.01	0.005	84.84	0.004	84.27	0.004
*Panthera pardus*	C	202	0.034	158	0.039	312.1	0.041	314.4	0.044	305.3	0.038	311.3	0.039
	P2	227	0.027	188	0.034	367	0.036	371.9	0.041	358.1	0.036	365.6	0.035
	P3	283	0.027	208	0.036	415.9	0.039	426	0.037	406.3	0.035	418.6	0.042
	P4	360	0.027	254	0.037	496	0.045	524.5	0.044	490.7	0.038	510	0.045
*Procyon lotor*	C	74.5	0.011	74.6	0.012	85.84	0.013	88.02	0.014	83.3	0.014	84.19	0.015
	P1	52.9	0.01	81.4	0.012	94.26	0.013	97.22	0.014	89.57	0.014	90.08	0.014
	P2	85.3	0.011	85.8	0.012	101.2	0.013	103.6	0.014	97.29	0.014	97.52	0.014
	P3	59.6	0.01	96	0.012	109.8	0.013	111.6	0.013	106.7	0.014	108.4	0.014
	P4	106	0.01	105	0.011	126.4	0.012	123.9	0.013	120.6	0.013	117.3	0.013
	M1	140	0.009	121	0.01	144.1	0.012	145.9	0.012	139.3	0.012	137.4	0.012
* *	M2	140	0.009	144	0.01	168.3	0.011	173.2	0.011	169.7	0.012	164.7	0.012
*Thinocyon velox*	C	22.9	0.002	22.2	0.002	25.49	0.002	25.32	0.002	25.28	0.002	25.31	0.002
	P1	26.8	0.002	25.9	0.002	29.86	0.002	29.24	0.002	29.69	0.002	29.47	0.002
	P2	30.6	0.002	29.8	0.002	34.22	0.002	32.88	0.002	33.78	0.002	33.31	0.002
	P3	35.5	0.002	34.2	0.002	39.3	0.002	38.94	0.002	39.01	0.002	39.27	0.002
	P4	39.8	0.002	38.4	0.002	44.45	0.002	44.77	0.002	44	0.002	44.57	0.002
	M1	46.8	0.002	45.2	0.002	52.73	0.002	52.92	0.002	52.82	0.002	52.94	0.002
* *	M2	56.1	0.002	54	0.002	62.84	0.002	62.59	0.002	62.24	0.002	62.94	0.002

BF: bite force (in Newtons), SE: strain energy (in Joules). Lores, Medres, and Hires models correspond roughly to 500k, 1,000k, and 1,500k tetrahedral elements, respectively. *Canis lupus* input force: 2509.99 N, volume: 207926 mm^3^, specimen LACM(M)23010. *Herpestes javanicus* input force: 262.21 N, volume: 10224 mm^3^, specimen AMNH(M)101655. *Mephitis mephitis* input force: 217.42 N, volume: 8856 mm^3^, specimen AMNH(M)172133. *Oodectes herpestoides* input force: 262.08 N, volume: 7947 mm^3^, specimen AMNH140008. *Panthera pardus* input force: 2168.94 N, volume: 40912 6mm^3^, specimen AMNH(M)113745. *Procyon lotor* input force: 650.91 N, Volume: 37352 mm^3^, specimen AMNH(M)24815. *Thinocyon velox* input force: 261.51 N, Volume: 11658 mm^3^, specimen FMNH PM60215. Seven bite positions were simulated for all species except for two species that have lower tooth counts: *Mephitis mephitis* (5 bite positions) and *Panthera pardus* (4 bite position). Institutional abbreviations: AMNH, American Museum of Natural History; FMNH, Field Museum of Natural History; LACM, Natural History Museum of Los Angeles County; M, Mammalogy collection.

**Table 3:** Scaling relationships of FEA results.

	Position	Regression Equation	r^2^	RMA slope	Isometry slope	Pearson Product-Moment Corr. Coef.	*p*	Scaling
Input Load-Bite Force	Canine	*y* = 0.2268*x*+2.3092	0.9817	1.02	1	0.9903	**<0.0001** *	Isometry
	Last tooth	*y* = 0.1082*x*+3.7178	0.9768	1.04	1	0.9842	**<0.0001** *	Isometry
Volume-Input Load	all	*y* = 0.0057*x*+342.57	0.7787	0.51	0.66	0.6721	0.05	-
	w/o *Panthera*	*y* = 0.0114*x*+156.06	0.9981	0.49	0.66	0.458	0.18	-
Volume-Strain Energy	Canine	*y* = 2x10^-7^ *x+*0.0092	0.3714	0.74	1	0.6177	0.07	-
	Last tooth	*y* = 1x10^-7^ *x+*0.0076	0.4298	0.75	1	0.6491	0.06	-
w/o *Panthera*	Canine	*y* = 5x10^-7^ *x*-0.003	0.9972	0.79	1	0.4316	0.2	-
	Last tooth	*y* = 5x10^-7^ *x*-0.0026	0.9965	0.78	1	0.4569	0.18	-
Input Load-Strain Energy	Canine	*y* = 8x10^-7^ *x* ^1.4462^	0.9598	1.464	1.5	0.9806	**<0.0001** *	Negative allometry
	Last tooth	*y* = 9x10^-7^ *x* ^1.4436^	0.952	1.4807	1.5	0.9855	**<0.0001** *	Negative allometry
w/o *Panthera*	Canine	*y* = 4x10^-7^ *x* ^1.6065^	0.9708	1.6107	1.5	0.9832	**<0.001** *	Positive allometry
	Last tooth	*y* = 4x10^-7^ *x* ^1.5731^	0.9676	1.5788	1.5	0.9824	**<0.001** *	Positive allometry
Bite Force-Strain Energy	Canine	*y* = 2x10^-5^ *x* ^1.4244^	0.9528	1.4348	1.5	0.9838	**<0.0001** *	Negative allometry
	Last tooth	*y* = 8x10^-6^ *x* ^1.4099^	0.9545	1.4271	1.5	0.9844	**<0.0001** *	Negative allometry

Regression analyses were conducted on both raw values and phylogenetic independent contrasts. Homologous canine positions and analogous most posterior tooth positions were tested.

*Statistically significant result at *p* = 0.001.

**Fig 3:**
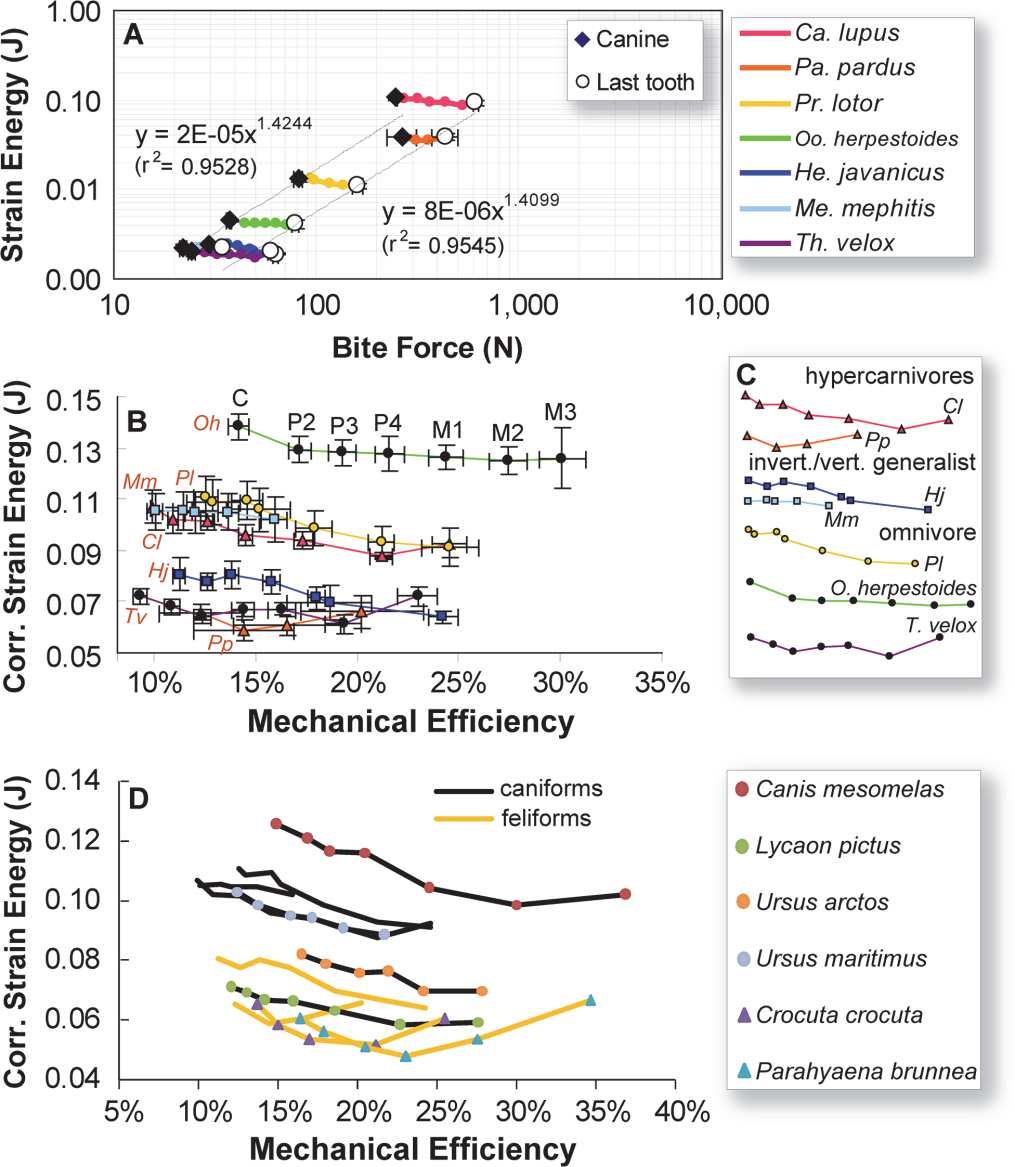
Biting biomechanics profiles of all skull models. **A**. Strain energy versusoutput bite force. **B**. Corrected strain energy versus mechanical efficiency, with strain energy values of each sampled species *x*, adjusted relative to *C*. *lupus* (“*x adjusted”*) using the equation: SE_*x*,*adjusted*_ = (Volume_*x*_/Volume_*C*.*lupus*_)^1/3^(InputLoad_*C*.*lupus*_/InputLoad_*x*_)^2^*SE_*x *_[28]. **C**. ME-SE profiles from part B categorized according to ecomorphs, with x and y axes representing relative magnitudes of mechanical efficiency and corrected strain energy, respectively. **D**. ME-SE profiles of the 5 extant species models constructed in this study (marked as caniform or feliform by line color only, no point labels), plus 6 additional species models taken from the literature (marked by circles for caniform and triangles for feliform species) [39,40,44,45]. Note general separation between caniform and feliform curves, the one exception being *Lycaon pictus*. Abbreviations as in Fig 2.

FE analyses of the species models indicated a power relationship between bite force and skull strain energy values. Increases in bite force, which are correlated with increases in overall size, correspond to increasing skull strain energy with an exponent of 1.41–1.42. This is expected, in part, because of the isometric scaling relationship of strain energy proportionately to volume and input force squared [28]. However, a positively allometric relationship is present for bite force relative to strain energy (or strain energy being negatively allometric relative to bite force) in both regressions of raw values and PICs (Fig 3A and Table 3). The *Canis* model has lower bite forces than expected, and the *Panthera* model has higher canine bite force than predicted by the regression line across all species sampled (Fig 3A).

After bite force was adjusted for model input force (by calculation of mechanical efficiency, or output bite force divided by input muscle force) and strain energy adjusted for volume (by multiplying expected allometric relationships to volume and surface area using the equation SE_*x*,*adjusted*_ = (Volume_*x*_/Volume_*C*.*lupus*_)^1/3^(InputLoad_*C*.*lupus*_/InputLoad_*x*_)^2^*SE_*x*_ from [28] which account for both volume and input force differences as a result of size difference) to remove size effects, respectively, the seven models fell into three groups (Fig 3B): a high strain energy *Oodectes* model, three medium strain energy models (the caniforms *Procyon*, *Mephitis*, *Canis*), and three low strain energy models (the feliforms *Herpestes* and *Panthera*, and the outgroup taxon *Thinocyon*). The three strain energy groupings are significantly different based on the lack of overlap among the ranges of uncertainty in the models (one standard error in each direction) across the three groups (Fig 3B). These groupings differentially cluster taxa of caniforms (medium strain energy models) and feliforms (low strain energy models), representing the two major clades within Carnivora, with the sole basal carnivoramorphan sampled exhibiting a high strain energy model and the outgroup clustering with the feliforms in having the most work-efficient (lowest strain energy) models. Although neither adjusted strain energy (SE) nor mechanical efficiency (ME) values correspond with the dietary-feeding categories, the shape of the ME-SE curves across the dentition does split into two major feeding types among the sampled species. The curves for invertebrate/vertebrate generalists (*H*. *javanicus*, *M*. *mephitis*) and an omnivore (*P*. *lotor*) tend to decrease towards the back tooth positions, whereas those of hypercarnivores (*C*. *lupus*, *P*. *pardus*) tend to be concave, with the low point (i.e. lowest strain energy bite position) towards the middle or rear of the dentition (Fig 3C).

**Fig 4:**
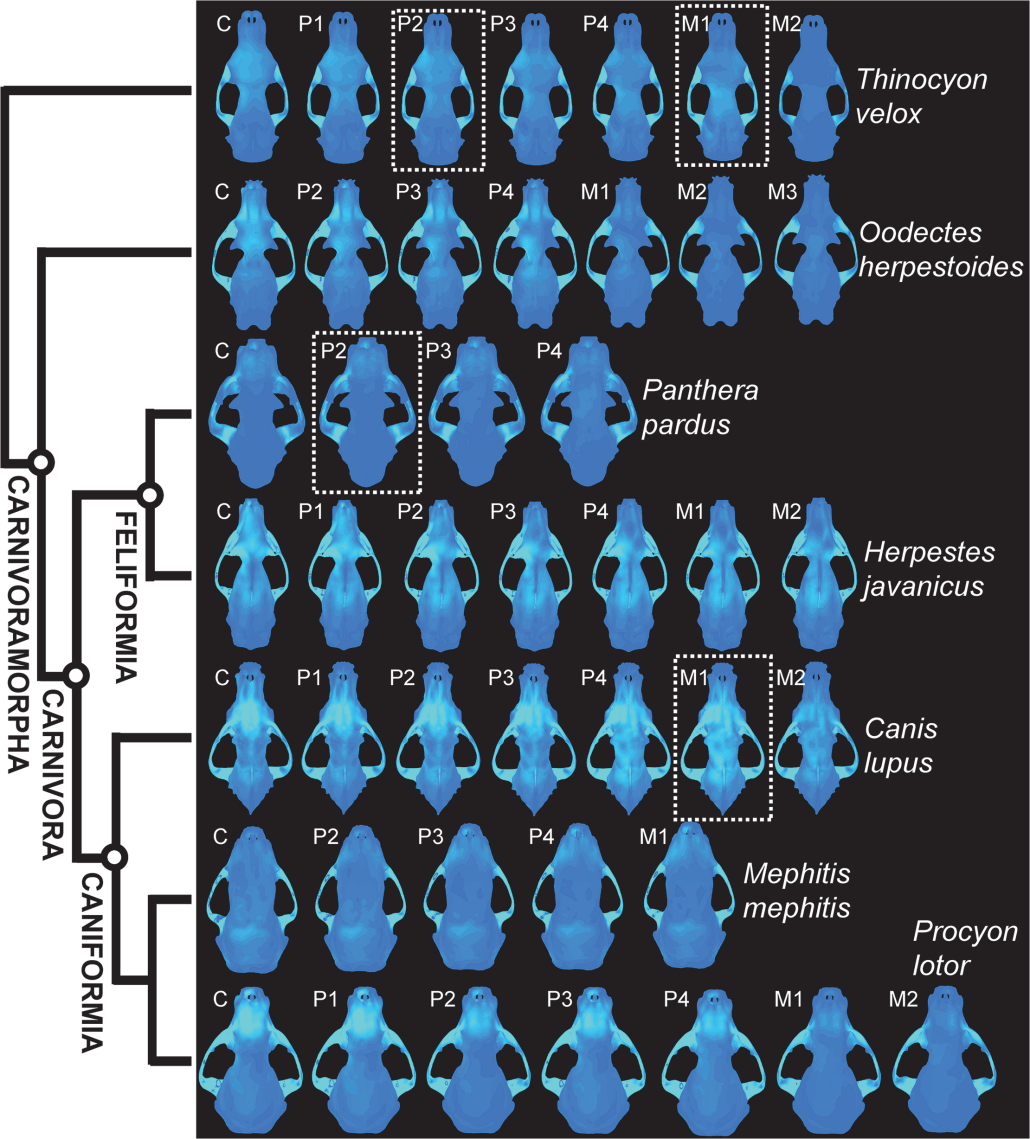
von Mises stress distributions across bite positions in different species. Visualized colors are standardized to 10% of max von Mises stress values in each bite analysis of every skull model to show the distribution of the most widespread stress locations. Elevated stress values are indicated by lighter blue color, whereas low or no-stress regions are in darer blue. Dotted boxes indicate bite positions in hypercarnivores that show optimal ME-SE ratios, as in Fig 3C.

Distributions of von Mises stress in the cranium under different bite loci simulations do not show clear distinctions between the feeding categories (Fig 4). The extant invertebrate/vertebrate generalist *Herpestes javanicus* and the omnivore *Procyon lotor* exhibit a trend of decreasing von Mises stress in the skull from the anterior to the posterior tooth locus bite simulations; the extant hypercarnivore *Panthera pardus* and invertebrate/vertebrate generalist *Mephitis* show similar stress distributions regardless of bite positions (Fig 4). The trend for the stem carnivoramorphan *Oodectes herpestoides* is more similar to those for *Herpestes* and *Procyon*, whereas the trend in the creodont outgroup taxon *Thinocyon velox* is more similar to those of *Panthera* and *Mephitis*. The skull of *Canis lupus* shows the most widely distributed cranial stress in the P4-M1 biting simulations, exhibiting lower stress in the anterior dentition, and lowest stress at the last tooth position (M2).

### Geometric morphometric analyses

**Fig 5:**
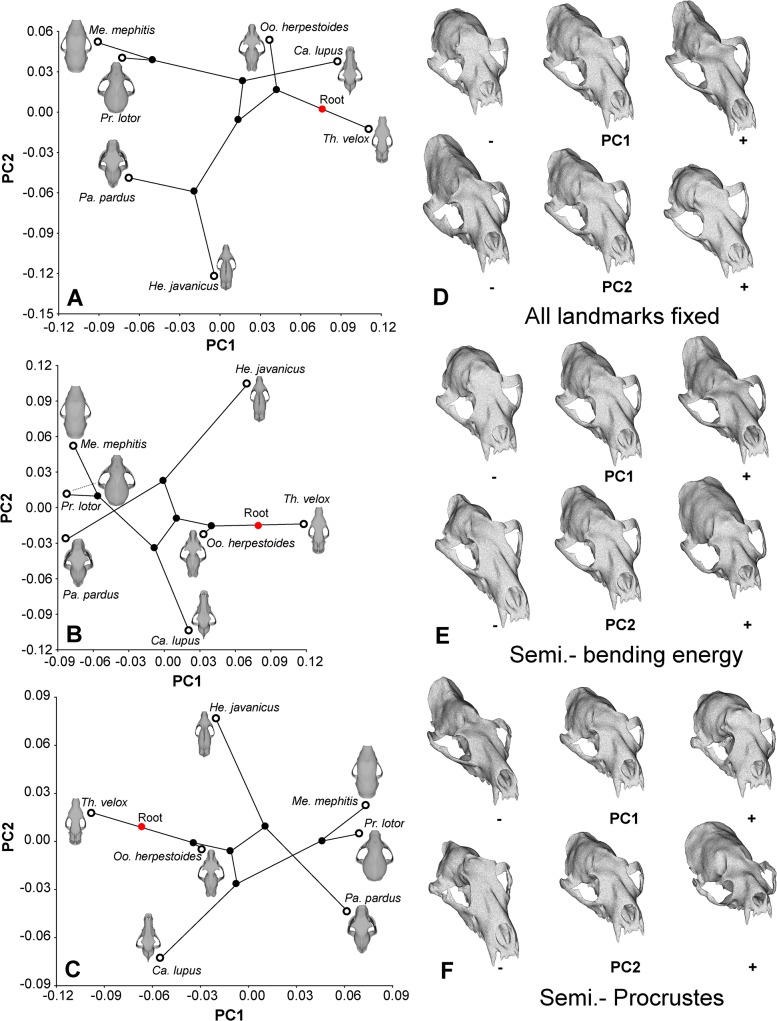
Phylomorphospace derived from GMM analyses and major directions of cranial shape change shown using morphed skulls of the average shape among species studied. **A**. PC1-2 plot of fixed landmark analysis. **B**. PC1-2 plot of sliding semi-landmarks analysis with bending energy superimposition. **C**. PC1-2 plot of sliding semi-landmarks analysis with Procrustes superimposition. **D**. Directions of skull shape change along the first two PC axes in the fixed landmark analysis. **E**. Directions of skull shape change along the first two PC axes in the semi-landmarks analysis with bending energy superimposition. **F**. Directions of skull shape change along the first two PC axes in the semi-landmarks analysis with Procrustes superimposition.

Principal components analysis of the size-corrected landmark shape variables generated five orthogonal PC axes. The first three PC axes accounted for 86.40% of total variance (PC1: 45.29%, PC2: 30.02%, PC3: 11.09%). The same shape data, analyzed by first optimizing the superimposition of the semi-landmarks over the temporalis and masseter regions, using either a Procrustes method (first 3 PCs: 81.88%) or a minimized bending energy method (first 3 PCs: 83.08%), both returned lower amounts of variance explained by the each of the first three PC axes (Fig 5: PC3 not shown). After mapping shape variables onto the phylogenetic tree, a permutation test failed to reject a null hypothesis of no phylogenetic signal in the dataset (1 x 10^6^ iterations, *p* = 0.1336). In addition, permutation tests did not detect a phylogenetic signal in shape variation relative to centroid size (*p* = 0.6610). For ease of comparison and presentation, only results of the first two PCs are plotted, as these axes accounted for over 70% of total variance, and therefore captured the major trends among the skull shapes.

In the fixed landmark analysis, PC1 separated dorsoventrally compressed and anteroposteriorly elongate skulls with more anterior temporalis (longer input arm) and more posterior masseter (shorter input arm) areas relative to the temporomandibular joints (TMJ) at the positive end (*Oodectes*, *Canis*, *Thinocyon*), from more compact and deep skulls at the negative end (*Mephitis*, *Procyon*, *Panthera*). *Herpestes* occupies an intermediate position on this PC axis. PC2 separated skulls with anteroposteriorly short, dorsally positioned zygomatic arches (suggesting smaller area for the masseter muscles and less effective input force) and a down-turned rostrum at the positive end (*Mephitis*, *Procyon*, *Canis*, *Oodectes*, *Thinocyon*), from skulls with more dorsally located rostrum relative to the braincase, and more anteroposteriorly extended, deeper and ventrally positioned zygomatic arches (*Panthera*, *Herpestes*) towards the negative end. PC3 separated skulls with more vertically oriented temporalis areas (*Mephitis*, *Panthera*, *Thinocyon*, *Canis*) at the positive end, from skulls having more anteroposteriorly oriented temporalis regions (*Herpestes*, *Oodectes*, *Procyon*) at the negative end (Fig 5A and 5D shown for PC1-2).

Analyses implementing sliding semi-landmarks at the temporalis and masseter regions indicated broadly similar orthogonal axes of shape change. Compared to the fixed landmark analysis, which showed changes in overall skull length in PC1 and relative shortening of the braincase in PC2, the sliding semi-landmark analyses yield more relative changes between the rostrum and braincase in PC1 and PC2. In the sliding semi-landmark analyses, PC1 captures relative areas of the temporalis regions, with *P*. *pardus*, *M*. *mephitis*, and *P*. *lotor* having more bulbous braincases than others. PC2 in the sliding semi-landmark analyses tends to be associated with increases in proportions of the braincase relative to the rostrum, with *H*. *javanicus* and *P*. *lotor* having proportionally larger braincase and temporalis areas than other species (Fig 5B, 5C, 5E and 5F. The hypercarnivores are separated from the invertebrate/vertebrate generalists and omnivores along PC2 in the sliding semi-landmark analyses, but not in the fixed semi-landmark analysis (Fig 5).

Regression analyses of skull shape, represented by 3D landmark variables, and biomechanical parameters modeled by FE analysis (using canine bite force: input force and adjusted strain energy values as homologous points of comparison) returned statistically non-significant relationships (*p* = 0.69). Nevertheless, there is a clear trend of shape changes associated with bite force ratios; species with higher mechanical efficiency (bite force: input force ratios) tend to have a shorter rostrum, deeper and anteroposteriorly more elongate temporalis regions, and more anterodorsally positioned and deeper masseter regions on the zygomatic arches. On the other hand, species with higher strain energy values (i.e., less work-efficient skulls) tend to have more anteroposteriorly restricted temporalis regions, more posterodorsally situated zygomatic regions, shallower temporalis and masseter regions, and a more elongate and ventrally positioned rostrum relative to the braincase.

Regression analyses of ME and SE in canine and last tooth biting simulations relative to each of the five PC axes of skull shape variation also returned statistically non-significant relationships (*p* = 0.14–0.55), and bivariate plots showed no visible trends except for strain energy correlating with PC2 (higher energy correlating with more positive PC2 values), and bite force with PC3 (higher bite force correlating with lower PC3 values; data not plotted), suggesting that the majority of variation observed in the phylomorphospace (PC1 and PC2) is not significantly correlated to the two principal biomechanical attributes estimated by FEA. The phylomorphospace indicates branching and filling of unique regions in the morphospace, except for crossing of the branch leading to *Panthera* over that uniting the caniforms, with more negative PC2 values in the two hypercarnivores (*Panthera* and *Canis*) (Fig 5B and 5C). Furthermore, regressions of PC scores to biomechanical variables indicate skulls with lower strain energy values along PC2 have deeper zygomatic arches and larger masseter attachment areas, whereas skulls with higher bite force along PC3 have more expanded temporalis regions.

## Discussion

To test whether different feeding preferences can be distinguished by distinct biomechanical properties of the cranium, and how such a diet-biomechanics linkage is related to cranial shape, we analyzed biomechanical capability and variation in skull shape for several carnivoran dietary categories (hypercarnivore, omnivore, and invertebrate/vertebrate generalist feeder categories), with the goal of establishing a reliable diet-biomechanics linkage model to interpret the paleobiology of an exemplar stem carnivoramorphan and an outgroup creodont species. Results based on biomechanical simulations of biting in extant taxa provide a basis for confidently linking skull biomechanics with feeding preferences, but also indicate the presence of multiple sources of variation in biomechanical capability that could not be attributed to adaptation for a given diet alone.

### Allometric effects on biomechanical variables

FE analyses documented that estimated mechanical efficiency in the skull models were positively allometric relative to total strain energy (corresponding to negative allometry of total strain energy relative to mechanical efficiency increase), suggesting that mechanical efficiency increased faster than expected given the level of work-efficiency of the skull. The increase in bite force/input load is significantly correlated to skull centroid size with phylogeny taken into account (*p*<0.01), suggesting that the observed trend is size-driven. Positive allometry of estimated bite force relative to body size is observed in other vertebrates, such as during ontogenetic size increase of horn sharks [36]. As an analogous comparison, in horn sharks there is a concurrent increase in estimated cross-section area of the masticatory muscles and the mechanical advantage of the input-output lever arms contributed to allometric increase in more efficient force production in posterior bite positions, but for anterior bites the mechanical advantage scaled isometrically. In contrast, we found in the current study that mechanical efficiency is largely conserved, both at the canine and the last tooth bite position (and by extension all tooth positions in between) for all the species examined. The positive allometry of bite force relative to strain energy is therefore explained by lower increases in strain energy, given an isometrically increasing force-producing masticatory system. In other words, the load-deflection curve for linear elastic materials suggests that allometric increases in bite force without increases in strain energy must be achieved by decreased deflection in the structure ([28]:Fig 1), and a corresponding increase in the stiffness slope of the curve. Larger skulls thus are stiffer than expected by simple scaling relationships. Of course, such a conclusion is dependent on a simplified assumption of invariant bone type and distribution across species; an anatomical study to describe variation in cranial bone distributions in extant carnivorans is currently underway to further examine the potential influences of or sensitivity to bone variation. It is also worth noting that the majority of the significant correlations observed between biomechanical and morphological variables show comparably small deviations from isometry (Table 3).

### Morphology-biomechanics linkage

**Fig 6:**
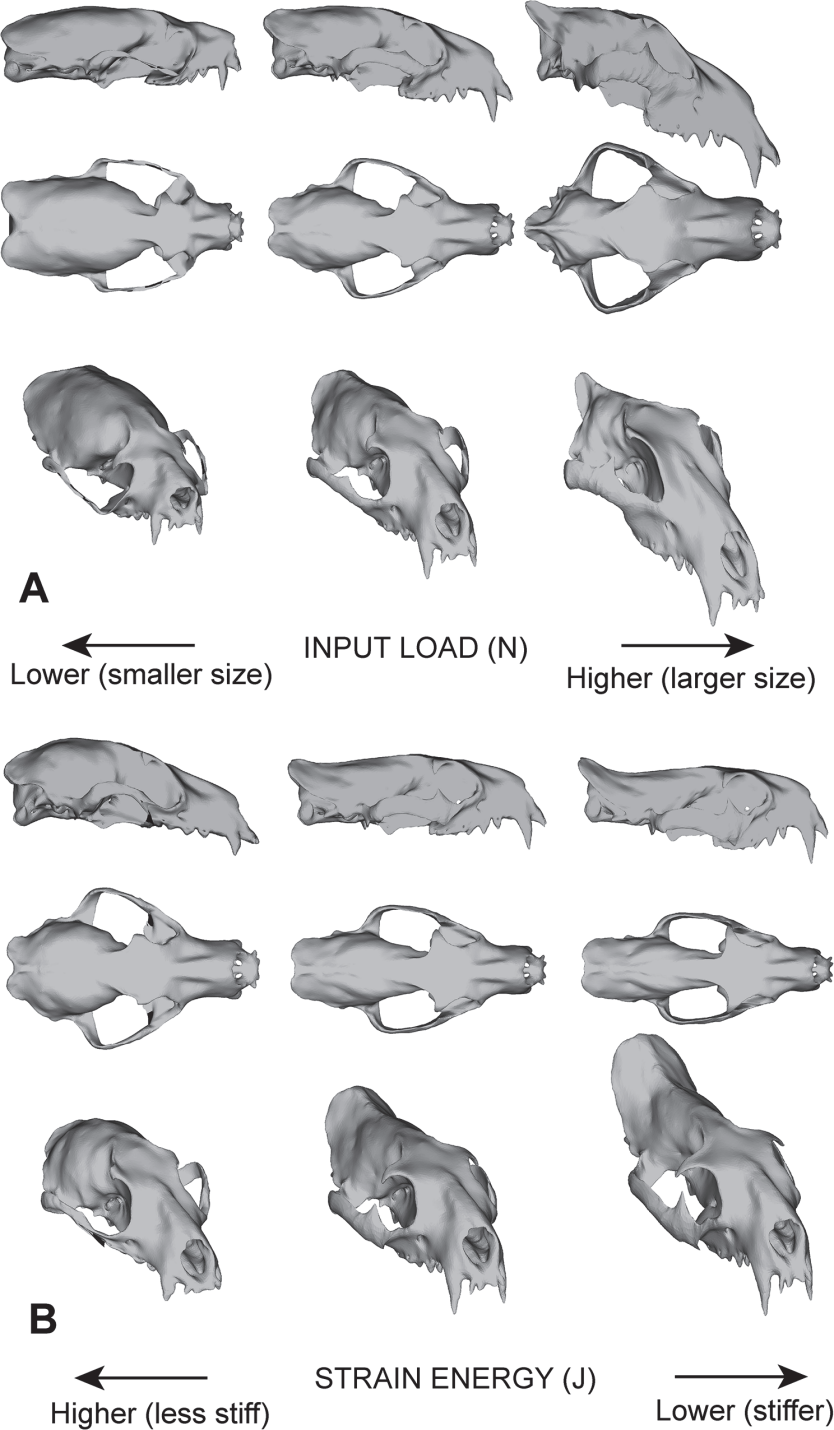
Cranial shape changes in morphed models, associated with A. input load / body size increase or decrease, and B. strain energy differences (higher or lower stiffness). Skulls were morphed from the model of *O*. *herpestoides* using trends from regression analyses of phylogenetic independent contrasts of input load, strain energy, and geometric skull shape.

In our analyses, size-based trends in biomechanical capability are correlated with changes in skull shape (albeit not statistically significant, *p* = 0.11), with larger species having a more dorsoventrally-arranged long axis through the rostrum and braincase, deeper muscle attachment regions, a more triangular dorsal skull profile, and broadening frontals (Fig 6). These rearrangements bring into alignment the long axes of the temporalis and masseter musculature with the dorsoventral axis of the skull, reorienting the effective force of the jaw-closing muscles into the dorsoventral direction, into the plane of jaw closure. A more vertically oriented skull also alters distribution of stress to a more dorsoventral direction (i.e. more compressive and less torsional forces) [14,37]. This change is consistent with mammalian cortical bone being strongest in compression, and not torsion or tension, and as a size-driven trend it cannot be uniquely correlated with specific feeding niches (e.g., large carnivorans such as ursids are omnivores and herbivores, whereas large canids and felids are hypercarnivores). Additional sources of variation, such as differences in distribution of cortical versus cancellous bone in the cranium, were not analyzed in this study. Differences in bone distribution and arrangement could contribute to the size-driven trends observed in this study, which held material properties constant in order to study the functional changes principally associated with skull shape differences.

### Phylogenetic effects on diet-biomechanics linkage

Once strain energy values were size-standardized using expected scaling relationships, and bite forces were compared as mechanical efficiency (ratio of input to output force), additional trends emerged from the profiles of output force to skull stiffness across the dentition. Mechanical efficiency, already shown by the regression analyses to be isometric, in addition shows a conserved range of values between 0.15–0.25 for all carnivorans and the outgroup (Fig 3B). The stem carnivoramorphan *Oodectes* stands out as having both elevated ME and SE values, suggesting a potentially unique feeding behavior. The weaker but more force-efficient skull, still constrained by the invariant ultimate strength of bone, may have been used to capture smaller prey in order to avoid overloading the skull, as has been proposed for crocodilians that have more slender rostral morphology than, but comparable mechanical efficiency to, robust species and thus are more prone to high bending moments during feeding [38]. It is possible that this observation for the extinct stem carnivoramorphan *Oodectes* represents a similar function or biomechanical behavior, which would indicate that such capability is a plesiomorphic or ancestral feature of all carnivoramorphans. This functional hypothesis requires additional species models to test whether an elevated ME-SE profile was a unique trait in *O*. *herpestoides*, or whether other stem carnivoramorphans also had fundamentally different cranial mechanical properties relative to crown carnivorans. It is also important to include and test additional outgroups to polarize any evolutionary trends in biomechanical adaptations. Lastly, unlike several previous studies that found a link between von Mises stress distributions and hunting or masticatory behavior [39,40,41], no such connection is observed in our dataset (Fig 4).

The distributions of modeled strain energy among extant species indicate that feliform skulls are stiffer (have lower SE values) than caniform skulls, even though we sampled both caniform and feliform hypercarnivores (*C*. *lupus* and *P*. *pardus*) and invertebrate/vertebrate generalists (*M*. *mephitis* and *H*. *javanicus*). This suggests that these two feeding categories cannot be differentiated using properties such as mechanical efficiency and strain energy alone, as might be expected based on observed differences in food choice and properties, and prey processing behavior [42,43]. Instead, species with lower corrected strain energy levels (both feliforms and the outgroup *T*. *velox*) tend to have deeper facial regions, deeper zygomas, and more prominent occipital crests, and an overall laterally compressed skull compared to caniforms (Fig 6). These morphological differences reflect a more tubular cranial form, and have ePCSA of jaw muscles that are closer to the mid-sagittal axis of the skull, effectively reducing the force moment arms and bending moments in the lateral direction.

As a “sensitivity” test of the observed feliform-caniform split in SE results, we ran the same set of FE analyses on six additional extant carnivoran species skull models from previous publications: *Canis mesomelas *[39], *Lycaon pictus *[40], *Ursus arctos* and *U*. *maritimus *[44], and *Parahyaena brunnea* and *Crocuta crocuta *[45] (modified versions of the published models are also available at the Dryad data repository, doi:10.5061/dryad.1b52s). In contrast to our own dataset, these six models are single models, not ones built using the resampling method adopted in this study to assess uncertainty range in the measured ME and SE values from FE analyses. We modified only the magnitude and directions of the applied muscle forces in those models using the protocol described in Materials and Methods. The resulting ME-SE plots followed the findings of our own dataset, with all additional caniform models but one having higher SE than both the originally analyzed and previously published sensitivity test feliform models (Fig 3D). There is no overlap between the ME-SE curves of caniform and feliform models, except for that of *Lycaon pictus*, which has SE values that fall within the upper end of the distribution of all the feliforms analyzed. We selected the African wild dog, *Lycaon pictus*, as an extreme example of a caniform species with feliform-like features. *Lycaon pictus* is a pursuit-hunting hypercarnivore, with a shorter and wider rostrum than in most other living canids, and relatively stiffer and more efficient cranial biomechanics [39]. In this case, the low SE values of the *Lycaon* bite simulations appear to reflect its almost exclusively hypercarnivorous diet and also its propensity to hunt large prey, all are features shared with some of hypercarnivorous felid and hyaenid feliforms analyzed here. The presence of higher-level taxonomic groupings in skull strain energy values in both the primary analysis of models/data analyzed with this new method, and in the sensitivity test applying a similar analytical approach to single previously published cranial models, warrants further study with a broader phylogenetic and dietary sample, but the persistence of this pattern in an expanded sample containing 11 species ranging across clades and with both overlapping and distinct dietary specializations (Fig 3D) suggests that comparisons of skull biomechanics between species using FE analyses need to account for phylogeny. Our future analyses of extant carnivoran species across all major clades, with both broader and deeper sampling among and within dietary categories, will enable further verification or potential rejection of this distinctive caniform-feliform separation in SE.

### Corrected diet-biomechanics linkage model

After the identification and accommodation of size allometry (via adjustment of FE analysis results to expected area and volume relationships) and phylogenetically correlated biomechanical traits (by not comparing strain energy magnitudes independent of mechanical efficiency), differences remaining in the shape of the ME-SE curves then reflect the major feeding categories used to characterize diet. These differences are used as the diet-biomechanics linkage models to tie simulation results to feeding preferences. The extant hypercarnivores exhibit a concave ME-SE profile, with highest stiffness (lowest SE) in the skull in the middle of the dentition. For *P*. *pardus* this position is just behind the canines, and for *C*. *lupus* it is behind the carnassials (Fig 3C). The relative optimization in stiffness in the two hypercarnivores can be related to their morphology and feeding behavior: felids have reduced cheek dentitions, and mainly use strong canine killing bites to suffocate and hold their prey and their posterior teeth for cutting soft tissues, whereas canids tend to utilize high molar bite forces to crush harder food items (as opposed to using hypertrophied premolars for cracking, as in hyaenids). The overall differences in the shape of ME-SE curves between hypercarnivores and dietary generalists are also reflected in the phylomorphospace generated using sliding semi-landmark methods, but not the analysis using the fixed landmark/semi-landmark method (Fig 5).

Given that the FE methods used in this study of skull biomechanical properties specifically simulated muscle-induced, “intrinsic” loads at the tips of each tooth locus, the interpretations and trends are most pertinent to differential abilities of the studied species to process or masticate food. Such a focus does not directly address the full range of food acquisition strategies that carnivorans utilize to catch prey in the first place (see [39,40,41] for simulations that address locomotion and prey-induced “extrinsic” loads), but the same phylogenetic and allometric influences should be present in the results of “extrinsic” load simulations, given the current protocol of adding twisting or pulling forces on top of already established fundamental intrinsic loads, to simulate the effects of extrinsic forces [39, 40]. Considering that carnivoran species with drastically different prey-acquisition strategies are still exposed to similar masticatory challenges when attempting to process food items of similar material properties (e.g., vertebrate soft tissue, vertebrate bone, insect exoskeleton, etc.) after prey capture/food gathering, we examined only these final-stage intrinsic forces by simulating food-processing with point loads, as an initial approach to finding broad connections between skull biomechanical variables and dietary preference. The complex interplay between modeled intrinsic loads, phylogeny, and size effects in this study indicates that analyses of extrinsic loads not only will need to estimate forces generated by hunting prey of different sizes relative to the carnivoran species modeled, but also must take into account the same factors that we discovered to influence intrinsic load simulations.

### Application of diet-biomechanics linkage model to extinct species

We next use the current morpho-functional linkage model to reconstruct feeding categories of the extinct near outgroup *T*. *velox*. The dentition of *T*. *velox* possessed two optimized positions for skull stiffness, at the anterior and posterior portions of the dentition. Such a bimodal specialization in biting efficiency is reminiscent of the caniniform and molariform teeth in *Alligator *[38], in which the anterior caniniform teeth are used for seizing prey, and the posterior molariform teeth are used for crushing and seizing prey. The corresponding morphological features for such a bimodal function in *Alligator* include dorsoventrally deepened rostral bone in the areas of the caniniform and molariform teeth. Without corresponding bimodal profiles in the extant carnivoran models analyzed, the ecological habits of *T*. *velox* could therefore only be interpreted as having some type of bimodal function, potentially similar to those observed in the extant long-snouted crocodilian generalists. The overall distributions of stress in the skull of *T*. *velox* at different bite positions show more similarity to *P*. *pardus* than to *C*. *lupus* (Fig 3); this may indicate capability for the use of killing bites in *T*. *velox*, and less reliance on shallow slashing bites as observed in the more stressed cranial model of *C*. *lupus* under unilateral biting simulations.

Besides the higher magnitude ME-SE profile of *O*. *herpestoides*, the overall shape of its biomechanical curve most closely resembles extant invertebrate/vertebrate feeders and omnivores (Fig 3C). In the invertebrate/vertebrate feeding species models, SE drops continuously from the anterior to the posterior tooth positions, accompanied by a steady increase in mechanical efficiency as predicted from first principles of lever mechanics. This SE profile indicates that skulls of invertebrate/vertebrate feeders and omnivores do not show differential mechanical response to different bite locations beyond what is expected from basic lever mechanics, and is consistent with the wide range of food items and material properties encountered in their diverse diets. The analyses conducted here, however, could not separate the more insectivorous (*H*. *javanicus* and *M*. *mephitis*) from the highly omnivorous (*P*. *lotor*) extant species. This may be an indication that generalist species, though they may differ in the proportions of food items regularly consumed in their diet, nevertheless have skulls that are generalized biomechanically with regard to types of food, but not their relative proportions in the diet (e.g. cranial shape distinguishes durophagous carnivorans, but not between bone and bamboo specialists: [46]). The trend of stress distributions in the skull of *O*. *herpestoides* is closest in both distribution and relative magnitude to that in *P*. *lotor* (Fig 3). The potential feeding preference of *O*. *herpestoides* would best be assigned to the generalist/omnivore category based on all available data, pending the illumination of better morpho-functional linkage models that can more finely characterize cranial mechanical properties within different generalist species, and sampling more taxa. Nevertheless, the presence of a generalist biomechanical profile in a stem carnivoramorphan species suggests that crown carnivoran generalists have similar mechanical profiles, at least in the shape of the ME-SE curve, to those that already were present in early stem species such as those in the *Oodectes-Vulpavus* clade more than 40 million years ago. Inferred hypocarnivorous or omnivorous dental morphologies in the earliest canids suggest that such biomechanical profiles could be symplesiomorphic (primitive) or ancestral for crown group Carnivora [47,48]. The interpretation of *Oodectes herpestoides* being a generalist and *Thinocyon* being a hypercarnivore suggests that such distinct categories of dietary preference had already evolved in or were ancestral for early Cenozoic Ferae (the clade containing Carnivoramorpha and creodonts [49,50]), prior to the origin of similar feeding strategies within crown carnivoran predator guilds.

### Implications for future research

More broadly, the fact that differences between ecomorphs emerged only when the ME-SE profile curves of the entire dentition are compared indicates that using FE analyses of single bite positions to compare species, as in many previous studies, will not capture finer details about overall skull biomechanics that are more clearly divided according to known feeding preferences when examined across the dentition. A disconnect between bite force generation in functionally similar tooth positions and feeding preference has been observed within felids [47], and the results from the current analysis indicate that a lack of correspondence between biting efficiency at a single tooth locus of comparison to feeding preference may be true for other carnivorans also. This observation supports the use of biomechanical profiles across the entire canine to molar dentition to more broadly characterize feeding niches.

Plotting the relationships among input muscle force, total strain energy, and total skull volume revealed that although *P*. *pardus* has relatively stronger anterior bite force for its skull work-efficiency (low strain energy), it does so not only by the positive size allometry present across carnivoran skulls, but also its high skull volume relative to ePCSA of jaw musculature (Fig 2). The relatively high volume of large felid skulls appears to be achieved by increased volume of cancellous bone relative to cortical bone [51], and larger felids have high bone volume relative to their skull surface area [52]. Interestingly, anatomical studies of felid species found positive allometry of estimated bite force and PCSA relative to body mass and cranial size [53], a trend that was non-significant in the current analysis for Carnivora, but potentially caused by the low statistical power of the small sample for rejecting the null model of no correlation. Given a rather conserved craniodental morphology in Felidae [54], it would be interesting to study more felid species of different sizes to determine if they generally fall along a different allometric trajectory in biomechanical attributes compared to broader carnivoran samples.

The overall trends in cranial shape changes along the input load and strain energy gradients indicate that, to some degree, the morphological correlates of size and input load increase is in conflict with strain energy patterns, the latter being associated with a feliform-caniform split (Figs 3 and 6). Whereas larger skulls with increased bite force tend to have wider zygomas, stiffer size-standardized skulls (e.g., those of feliforms) in the sample tend to have narrower zygomas (Fig 6). In other words, morphological changes in the stiffer feliform skulls associated with size change involve widening of the zygomatic breadth as a function of body size increase, which by itself decreases stiffness. This conflicting change in morpho-functional association indicates the presence of functional constraints, and tradeoff in size-associated versus phylogenetically-correlated morphological changes and further emphasizes the importance of identifying diet-biomechanics linkages that persist in spite of those influences.

## Conclusions

Biomechanical and shape analyses were conducted on extant carnivoran species with different feeding preferences to test the ability of simulation-based biomechanical proxies to accurately reconstruct feeding niches, and how such diet-biomechanics linkage models can inform inference of potential feeding preference in extinct species. Masticatory simulations of the canine to molar dentitions in extant carnivoran skull models and two extinct species showed that estimated maximum muscle force and output bite force are positively allometric relative to skull work-efficiency, with increases in size, and that larger species tend to have more dorsoventrally oriented skulls that are stiffer relative to the bite force produced. The range of mechanical efficiency in the dentition is conserved across carnivoran models, and skull stiffness is generally higher in feliforms than in caniforms regardless of feeding niche. Both are interpreted as plesiomorphic or ancestral biomechanical features at their respective clade levels. After accounting for these size-related and phylogenetic effects of cranial mechanical properties, the skulls of hypercarnivores are characterized by differential optimization of skull stiffness to force output at the anterior dentition for ambush killing felids, and at the posterior dentition for canids utilizing crushing bites. Both omnivores and invertebrate/vertebrate feeders have skulls that exhibit gradual increases in work efficiency and force output from the anterior to the posterior dentition, indicating generalized skull biomechanics corresponding to generalized diets.

The creodont outgroup species *Thinocyon velox* exhibits a bimodal ME-SE profile, indicating mechanical properties associated with both prey capture using anterior teeth and crushing using posterior teeth, and may represent a unique hypercarnivorous feeding style among the species examined. The stem carnivoramorphan *Oodectes herpestoides* has a biomechanical profile similar to extant generalists, but has an elevated ME-SE profile unlike any of the extant feeding categories analyzed, suggesting suitability for feeding on smaller prey relative to their body size compared to extant generalists. Because such biomechanically relevant proxies are directly linked to measures of performance for specific, ecologically important tasks, they have the potential to provide more tightly associated form-function linkages in extinct taxa than traditional linear multivariate morphological proxies of diet, for which functional significance is only broadly defined. The complex interactions of phylogenetic, body size, and ecological factors observed to be associated with specific sets of cranial mechanical properties suggest that the use of diet-biomechanics linkage models to assign taxa to pre-defined feeding categories, and to study the paleobiology of extinct species relative to extant forms, both require careful isolation of factors that might influence our ability to reliably identify the ecological signals relative to ancestry or size influences. Our findings also clearly demonstrate that cranial mechanical properties in extant species can reflect a combination of both ancestral (phylogenetic retentions) and ecological (adaptive) traits. Thus interpretations of diet and feeding preference in fossils need to first account for potentially confounding factors of morphological evolution (relative to hypotheses of adaptation), such as size scaling issues and ancestral retentions or constraints, even though these nevertheless may represent existing exaptations [55] for the form-function relationship being studied.
